# Soft, Sustainable, and Sensitive: Biopolymer-Based Hydrogels as Recyclable Temperature Sensors for Skin-Integrated Electronics

**DOI:** 10.1021/acsabm.5c01607

**Published:** 2025-11-13

**Authors:** David Naranjo, Juan Torras, Jose García-Torres

**Affiliations:** † IMEM-BRT Group, Departament d’Enginyeria Química, EEBE, 16767 Universitat Politècnica de Catalunya, C/Eduard Maristany, 10-14, Ed. I, Second Floor, 08019 Barcelona, Spain; ‡ Biomaterials, Biomechanics and Tissue Engineering Group, Department of Materials Science and Engineering, Escola d’Enginyeria Barcelona Est (EEBE) and Institute for Research and Innovation in Health (IRIS), Universitat Politècnica de Catalunya (UPC), 08019 Barcelona, Spain; § Barcelona Research Center in Multiscale Science and Engineering, Universitat Politècnica de Catalunya, 08019 Barcelona, Spain; ∥ CIBER en Bioingeniería, Biomateriales y Nanomedicina, CIBER-BBN, 50018 Zaragoza, Spain

**Keywords:** chitosan, agarose, PEDOT:PSS, hydrogel, sustainable and recyclable temperature sensor, skin electronics

## Abstract

The development of sustainable, soft, and recyclable materials for skin-integrated electronics is critical for advancing wearable health monitoring while minimizing electronic waste. Here, chitosan–agarose-based hydrogels integrated with poly­(3,4-ethylenedioxythiophene):polystyrenesulfonate (PEDOT:PSS) are fabricated as recyclable, biocompatible, and thermoresponsive materials for flexible temperature sensors. The hydrogels are synthesized using a green and easy process, forming interpenetrated dual networks that exhibit high water content, mechanical compliance, and enhanced electroconductivity. Morphological analysis reveals highly porous interconnected structures, while Fourier transform infrared spectroscopy confirms the successful incorporation of PEDOT:PSS. The hydrogels display high swelling capacity, tunable mechanical properties within the physiological range of human skin, and enhanced electrochemical performance. The temperature-sensing capability of the hydrogels demonstrates a negative temperature coefficient of resistance (TCR) of up to −1.5% °C^–1^, outperforming similar hydrogel-based sensors while maintaining stability over repeated thermal cycles. Importantly, the hydrogels can be disassembled, reprocessed, and reused for multiple sensing cycles without significant loss of performance, demonstrating true recyclability and supporting circular material use in soft electronics. The convergence of natural biopolymers with conducting polymers within these hydrogels provides a promising platform for developing eco-friendly, flexible bioelectronic devices, aligning with the requirements of sustainable materials science while addressing the need for high-performance, soft temperature sensors for wearable healthcare applications.

## Graphical abstract

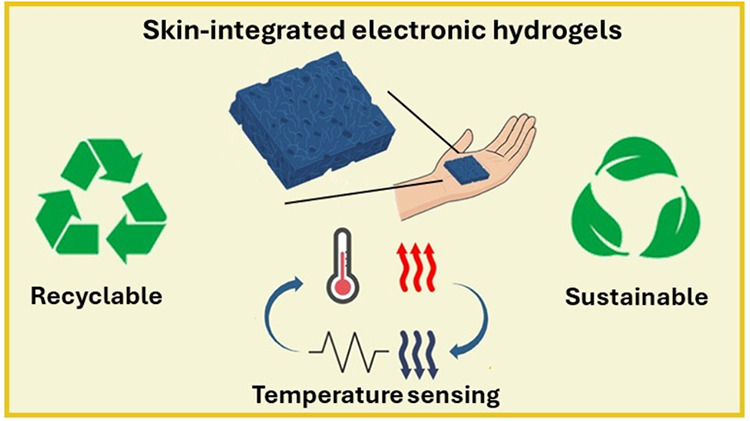

## 1 Introduction

Hydrogels—three-dimensional polymeric networks capable of absorbing and retaining large quantities of water—have attracted significant attention due to their unique combination of softness, flexibility, porosity, and high water content.^1,2^ These features render hydrogels particularly attractive for applications interfacing with biological systems, where mechanical compliance, hydration, and biocompatibility are essential.^3,4^ Despite their advantageous physicochemical properties, traditional hydrogels often lack functional responsiveness, which limits their performance in advanced technological and biomedical applications.^5,6^ This limitation has recently been addressed through the development of hydrogel metamaterials, an emerging class of materials in which hydrogels are engineered to incorporate additional properties—such as electrical conductivity, magnetism, and thermal sensitivity—enabling their use in high-performance applications ranging from drug delivery to bioelectronics and soft robotics.^7–10^

One of the most promising application domains for functional hydrogels is bioelectronics, a multidisciplinary field that aims to bridge the gap between electronic systems and living tissue.^11,12^ This interface requires materials that not only mimic the mechanical properties of biological systems but also offer electronic functionality. Traditional rigid and dry electronic materials like silicon or metal conductors are inherently incompatible with soft, wet, and dynamic biological tissues.^13,14^ In contrast, electrically conductive hydrogels (ECHs) provide an ideal platform: they combine the mechanical softness and water content of biological tissues with electronic conductivity, thereby enabling seamless signal transduction, sensing, and stimulation in implantable and wearable devices.^15,16^ The development of ECHs has been facilitated by the integration of conductive elements into hydrogel matrices. Various strategies have been employed, including the incorporation of metallic nanoparticles (e.g., gold, silver), carbon-based nanomaterials (e.g., graphene, carbon nanotubes), and—perhaps most significantl—conducting polymers (CPs).^17,18^ CPs such as polyaniline (PANI), polypyrrole (PPy), and poly­(3,4-ethylenedioxythiophene) (PEDOT) have emerged as particularly effective materials for conferring electrical conductivity to hydrogels due to their intrinsic charge transport capabilities, biocompatibility, and chemical tenability.^19,20^ Among them, PEDOT, especially in its doped form PEDOT:PSS, has garnered considerable attention due to its high conductivity, stability in aqueous environments, and compatibility with a wide range of biological systems.^21^

ECHs based on CPs have already demonstrated impressive performance in diverse applications: biosensors for real-time monitoring of metabolites and electrophysiological signals; actuators and artificial muscles capable of responding to electrical stimuli; and neural interfaces that record and stimulate brain activity.^22–24^ However, most of these devices rely on synthetic polymer matrices that, while effective, often lack biodegradability, reusability, and environmental sustainability—important criteria for next-generation bioelectronic devices, particularly those intended for transient or long-term implantation, or for use in resource-limited settings.^25^ To address these concerns, there is a growing interest in developing biopolymer-based conductive hydrogels. Natural polymers such as alginate, hyaluronic acid, chitosan, and agarose offer intrinsic advantages including biocompatibility, biodegradability, and renewable origin. These materials can serve as effective scaffolds for the incorporation of functional components, enabling the design of eco-friendly, recyclable, and even reprocessable bioelectronic systems.^26,27^ Importantly, their gelation properties and interaction with CPs can be finely tuned through chemical or physical cross-linking, thereby enabling controlled conductivity, mechanical compliance, and responsiveness.^28,29^

However, the main drawbacks of biopolymers are their weak mechanical properties.^30^ In this context, we have recently developed a dual biopolymer hydrogel with outstanding mechanical properties based on chitosan and agarose as compelling building blocks.^31^ Chitosan, a cationic polysaccharide derived from chitin, exhibits antibacterial activity, pH sensitivity, and mucoadhesiveness, making it suitable for biomedical and environmental applications.^32^ Agarose, a thermoresponsive polysaccharide extracted from red algae, exhibits reversible sol–gel transitions and forms highly porous, biocompatible networks.^33^ Their combination yields a hydrogel matrix that is not only mechanically robust and biocompatible but also suitable for functional modification. Thus, Viteri et al. successfully modified chitosan/agarose hydrogels with magnetite nanoparticles to wirelessly tailor drug release using external magnetic fields.^31^ Thus, incorporating PEDOT into such a biopolymer matrix enables the fabrication of multifunctional hydrogel metamaterials that combine the advantages of natural polymers with the high conductivity and electrochemical activity of CPs. Moreover, the resulting material can serve as the basis for temperature-responsive bioelectronic sensors, as temperature variations can modulate both ionic mobility and electronic properties within the hydrogel. This is particularly valuable in medical diagnostics and continuous monitoring applications, where local temperature fluctuations are indicative of physiological or pathological states (e.g., inflammation, infection, or tissue healing). Furthermore, the biopolymer-based composition of these hydrogels allows for recyclability and reusability—a key feature in the pursuit of sustainable electronics. Reversible gelation, water-based processing, and minimal cytotoxicity allow these materials to be repeatedly reshaped, rehydrated, and refunctionalized without significant degradation of performance. This recyclability makes the materials suitable not only for environmentally conscious applications but also for cost-sensitive deployments in disposable medical sensors or temporary wearables. Thus, in this work, the convergence of natural biopolymers (chitosan and agarose) with conducting polymers (PEDOT) opens a promising route to next-generation soft materials for bioelectronic applications. These chitosan/agarose/PEDOT hydrogels (Chit/Ag/PEDOT:PSS) combine sustainability, conductivity, and thermal sensitivity, making them ideal candidates for recyclable temperature sensors capable of interfacing with biological environments like skin. This work explores their synthesis, characterization, and performance in sensing applications, establishing a new class of functional hydrogels at the intersection of green chemistry, materials science, and bioelectronics.

## 2 Materials and Methods

### 2.1 Materials

Chitosan (molecular weight: 600,000–800,000) was acquired from Acros Organics, agarose (molecular weight: 306.12 g mol^–1^, Reference: BP160–100) was obtained from Fisher, poly­(3,4-ethylenedioxythiophene)-poly­(styrenesulfonate) (PEDOT:PSS) suspension (1.3%) was commercially available from Sigma–Aldrich. All products were used as received without further purification. All solutions were prepared using distilled water.

### 2.2 Chitosan-Agarose Hydrogel Synthesis

Chitosan-agarose hydrogels were prepared using a previously described procedure.^31^ Briefly, 100 mg of chitosan was dissolved in 5 mL of an acetic acid solution (0.5% v/v) at 25 °C. Simultaneously, 135 mg of agarose was dissolved in 5 mL of sodium hydroxide (0.1% w/v) at 85 °C. After that, both solutions mixed at constant stirring for 10 min while temperature was kept constant at 85 °C to avoid agarose gelation. Once both solutions were homogeneous, 5 mL of water were added drop by drop to reduce viscosity and favor the release of air bubbles entrapped in the chitosan-agarose solution. The resulting mixture was poured into a Petri dish (Ø = 10 cm), let it cool down until gelling at room temperature (4 h) and stored overnight (12 h) in the fridge (4 °C). Finally, the gel was dried at room temperature (∼25 °C) for 2 days, and the resultant film was carefully peeled off from the Petri dish.

### 2.3 Chitosan-Agarose-PEDOT:PSS Hydrogel Synthesis

The synthesis of chitosan-agarose-PEDOT:PSS hydrogels was similar as before, but chitosan was directly dissolved in the PEDOT:PSS dispersion as it has an acidic pH. To prepare hydrogels containing 5, 10, 20, and 30 wt % of PEDOT:PSS (relative to the total mass of solids in the hydrogel), a different volume of PEDOT:PSS commercial dispersion (1.3% w/v) was taken such that the required mass of PEDOT:PSS solids was obtained (see Table S1, Supporting Information). Chitosan was dissolved directly in this volume of PEDOT:PSS, and then distilled water was added to complete a total volume of 5 mL. In this way, the same final volume was maintained as in the control sample without PEDOT:PSS. Once chitosan, agarose and PEDOT:PSS were homogeneously mixed, the resultant solution was poured in the Petri dish, kept at room temperature for 4 h, kept overnight (12 h) in the fridge (4 °C) and back at room temperature (∼25 °C) for 2 days until total evaporation of water.

### 2.4 Characterization

Fourier transform-infrared (FTIR) spectroscopy was performed using a Jasco FTIR-4100 spectrophotometer at a resolution of 4 cm^–1^ in absorbance mode. Freeze-dried samples were analyzed with an attenuated total reflection (ATR) accessory featuring thermal control and a diamond crystal (Golden Gate Heated Single Reflection Diamond ATR). The absorption spectra were recorded after 64 scans in the 600–4000 cm^–1^ range, with automatic baseline correction applied via JASCO Spectra Manager software, version 2.

Scanning electron microscopy (SEM) was employed to study the morphology and porosity of the film samples. A Focused Ion Beam Zeiss Neon 40 SEM, operating at 5 kV, was used with a secondary electron detector to capture the micrographs. The freeze-dried samples were mounted on double-sided adhesive carbon discs and coated with a 10 nm layer of gold using a Leica EM ACE600 sputter coater to avoid sample charging. Pore segmentation was performed by adaptive thresholding (block size = 51 pixels, offset *C* = 10) with inverted binary mode, using Python/OpenCV. This binarization method allows robust detection of pores with variable contrast across the SEM images. Contours smaller than 5 pixels^2^ were discarded as noise. All measurements were subsequently scaled using the image scale bar (59 px = 200 nm).

The equilibrium swelling ratio (ESR, in %) of the films was measured by immersing samples of the dried films (*w*_d_) in 10 mL of Milli-Q water for 24 h at room temperature. Afterward, the swollen films were removed from the vials, excess water was removed, and the samples were reweighed (*w*_s_). The ESR was then determined using eq 1.

1 $$ESR\;\left(\%\right)=\frac{w_{s}-w_{d}}{w_{d}}\times 100$$

Biodegradation tests were conducted over a period of 9 weeks. Samples were initially freeze-dried and weighed (*w*_0_). Sterilization was performed by adding 1 mL of 70% ethanol to each well containing the samples for 30 min, followed by rinsing with phosphate-buffered saline (PBS). The samples were then submerged in 2 mL of PBS and incubated at 37 °C. Liquid lost to evaporation during the incubation was replenished as necessary. Following incubation, samples were washed thrice for 10 min with autoclaved distilled water. Samples were freeze-dried again, and their final weight (*w*_f_) was measured. Weight loss (*W*_l_) was calculated using eq 2.

2 $$W_{l}\;\left(\%\right)=\frac{w_{0}-w_{f}}{w_{0}}\times 100$$

Tensile tests were conducted using a Discovery HR-2 hybrid rheometer (TA Instruments, USA) equipped with a film tension clamp fixture. Hydrogel films were allowed to swell in deionized water for 24 h at room temperature prior to testing, then cut into uniform rectangular strips (4 cm × 0.8 cm). All tests were performed in triplicate at room temperature. A constant linear extension rate of 16.6 μm/s was applied until sample failure, and the resulting stress–strain curves were recorded.

The electrochemical properties were evaluated using cyclic voltammetry (CV) and electrochemical impedance spectroscopy (EIS) in a three-electrode setup with an Autolab PGSTAT302N. The films were fixed on the conductive side of an ITO glass which served as the working electrodes, with platinum coil as the counter electrode and Ag|AgCl as the reference electrode. Measurements were performed in 0.01 M PBS solution at room temperature. The potential range for CV spanned from −0.7 V to +0.7 V, with *a* scan rate of 100 mV s^–1^. Five consecutive oxidation–reduction cycles were applied. Electrochemical impedance spectroscopy (EIS) was performed using a Frequency Response Analysis (FRA) in potentiostatic mode at the open circuit potential (OCP) of each sample. A sinusoidal perturbation with an amplitude of 0.01 VRMS was applied, while the OCP values ranged between 17 and 176 mV vs Ag|AgCl electrode. The frequency range spanned from 1 × 10^5^ Hz to 0.1 Hz. The resulting impedance spectra were analyzed using Nyquist and Bode plots to assess the electrochemical properties of the films as *a* function of PEDOT content. Before electrochemical measurements, the films were submerged in PBS for 2 h to fully hydrate them.

To evaluate the temperature-sensing capability of the films, the relative change in electrical resistance as *a* function of temperature was measured. The upper portion of the swollen film was adhered to ITO film and connected to an Arduino Mega 2560 microprocessor. The system was set up as *a* potential divider with an input of 5 V supplied to the circuit. A Python program was developed to measure the resistance of the sample. The lower portion of the film was submerged in deionized water, and the system was gradually heated from room temperature to 50 °C. Temperature was controlled by *a* hot plate with thermocouple feedback: for each set point, the water was allowed to reach the target temperature and, once the thermocouple reading stabilized, we waited 10 min to ensure thermal equilibration between the water bath and the sensor. After that stabilization period, the signal (resistance) was recorded for 10 min, and the mean resistance over the recording interval was used as the steady-state resistance at that temperature. This procedure was repeated for all temperature points. The normalized resistance was calculated according to eq 3, and the temperature coefficient of resistance (TCR) was computed according to eq 4

3 $$\mathrm{normalized}\;\mathrm{resistance}=\frac{\Delta R}{R_{0}}=\frac{R_{0}-R_{T}}{R_{0}}$$

4 $$\mathrm{TCR}\;\left(\%\;{}^{\circ}C^{-1}\right)=\frac{R_{T}-R_{0}}{R_{0}}\cdot \frac{1}{\Delta T}\cdot 100$$

where *R*_*T*_ represents the real-time resistance measured at a temperature *T*, and *R*_0_ denotes the initial resistance at room temperature, *T*_0_. Furthermore, the temperature-sensing tests were carried out through cycles between room temperature and 50 °C on the Chit/Ag/PEDOT:PSS(20) and Chit/Ag/PEDOT:PSS(30) hydrogels.

The electrical performance of the hydrogels under compressive stress was also evaluated. Thus, controlled normal loads were applied to the hydrogel films. A glass vial with a contact area of 3.2 cm^2^ was gently placed on top of the film surface, ensuring uniform contact. Known masses of water were then incrementally added into the vial to produce pressures in the range of 0.15–0.61 kPa. After each mass addition, the system was allowed to stabilize before data acquisition. The resistance signal was subsequently recorded for 10 min under each pressure condition.

### 2.5 Film’s Recycling

Since agarose gels undergo reversible cross-linking, recycling assays were conducted to study the temperature-sensing stability of the film after dissolving and gelling it again. For this, after conducting the temperature-sensing tests on the sample, an amount of water equivalent to the evaporated mass after the drying process was added to the film and progressively heated up at 85 °C in a sealed Erlenmeyer flask with a septum stopper pierced by a needle for pressure relief. After complete homogenization, the synthesis process was followed as previously stated, and temperature-sensing tests were performed on the recycled samples.

## 3 Results and Discussion

### 3.1 Conceptualization, Design, and Fabrication of Hydrogels

Natural polymer-based hydrogels offer promising potential for bioelectronics, yet they still face several critical limitations.^26^ These include inadequate mechanical robustness, mismatch between the mechanical properties of the hydrogel and native tissues, nonsustainable or technically demanding synthesis routes, and limited recyclability—particularly when combining natural and synthetic components. In this work, we overcome these challenges by engineering an interpenetrating dual-network hydrogel composed of chitosan and agarose, since interpenetrated networks offer excellent mechanical properties, integrated with PEDOT:PSS to impart electric responsiveness. The resulting hydrogel is a soft, biodegradable, and recyclable electroconductive material with mechanical properties closely matching those of human skin. Chitosan, a cationic polysaccharide derived from chitin, is well recognized for its biocompatibility, biodegradability, antimicrobial activity, and processability in mildly acidic aqueous media. However, its inherent brittleness and poor mechanical stability limit its standalone use in load-bearing or mechanically dynamic environments. Agarose, a neutral polysaccharide extracted from red algae, complements chitosan by contributing a highly hydrated ECM (extracellular matrix)-mimetic network with favorable mechanical properties, oxygen and nutrient diffusivity, and structural stability. Their combination in a dual-network architecture not only addresses mechanical limitations but also enhances the hydrogel’s structural integrity. Although chitosan or agarose hydrogels have been tuned to display electrical conductivity,^34,35^ electroconductive chitosan-agarose hydrogels have not been fabricated before, and therefore there is much room for new applications. Thus, we have designed Chit/Ag/PEDOT:PSS hydrogels, that overcome previous limitations, for bioelectronic applications (e.g., temperature sensing). The hypothesis is that these hybrid systems will exhibit synergistic properties—mechanical compliance, electroconductivity, biofunctionality, recyclability—that are critical for emerging applications in soft bioelectronics. The present hydrogel system lays the groundwork for robust multifunctional platforms that can respond to electrical stimuli and are synthesized following an easy, green, scalable, and straightforward approach, relying on aqueous processing and mild conditions.

The overall synthesis procedure is depicted in Figure 1a. First, a chitosan solution was prepared by dissolving it the acid PEDOT:PSS dispersion at room temperature. Different amounts of PEDOT:PSS were added to have different contents into the final hydrogel (5, 10, 20, and 30% with respect total solid content) (Table S1, Supporting Information). Simultaneously, agarose solution was obtained by adding the biopolymer to a NaOH solution and heating it at 85 °C until agarose was fully dissolved. After that, the chitosan/PEDOT:PSS solution was slowly added into the agarose solution while keeping the temperature at 85 °C to avoid agarose gelation. Once a homogeneous mixture has obtained, it was poured in a Petri dish (Ø = 10 cm) and kept at room temperature for 4 h, followed by 4 °C overnight (12 h) to favor gelation and then left to dry at room temperature until a film was formed. Finally, the films were rehydrated to get the hydrogels. A blank chitosan-agarose hydrogel was also prepared following the previous protocol but chitosan was dissolved in an acetic acid solution instead of the PEDOT:PSS dispersion. Herein, hydrogels will be denoted as Chit/Ag, Chit/Ag/PEDOT:PSS(5), Chit/Ag/PEDOT:PSS(10), Chit/Ag/PEDOT:PSS(20) and Chit/Ag/PEDOT:PSS(30). Figure 1b show pictures of the synthesized films displaying varying concentrations of PEDOT:PSS, which were visually evident by a color change from colorless (in the pure Chit/Ag/PEDOT:PSS(0) hydrogels) to increasingly darker tones of blue as the PEDOT content increased. This color change indicates the successful incorporation of the conducting polymer into the hydrogel matrix. Despite the addition of PEDOT:PSS, all the hydrogels remained easy to manipulate, maintaining their flexibility and structural integrity, except Chit/Ag/PEDOT:PSS(30) hydrogel that was comparably more delicate to handle without breaking. These results will be confirmed and explained in the mechanical properties section.

**Figure 1:**
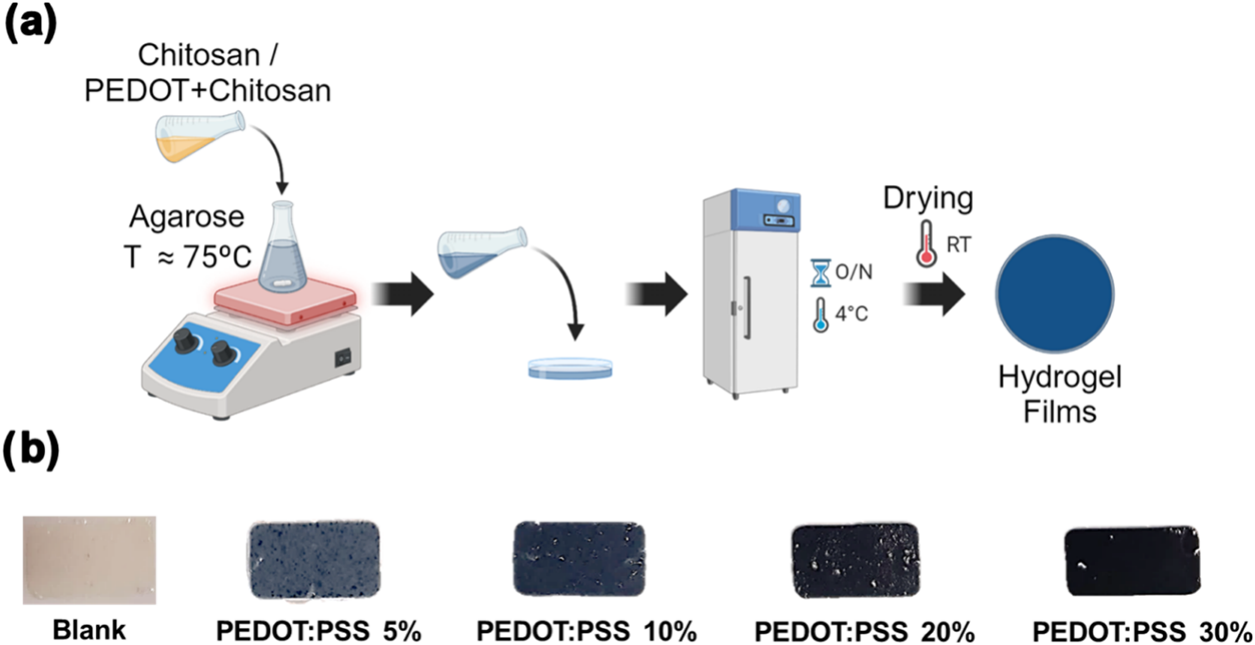
(a) Scheme of Chit/Ag/PEDOT:PSS hydrogels synthesis (Created in BioRender. Labay, C. (2025) https://BioRender.com/r74z537), (b) images of the different hydrogels fabricated: Chit/Ag, Chit/Ag/PEDOT:PSS(5), Chit/Ag/PEDOT:PSS(10), Chit/Ag/PEDOT:PSS(20) and Chit/Ag/PEDOT:PSS(30).

### 3.2 Morphological and Structural Characterization of Hydrogels

To gain insight into the microstructure of the materials, we performed scanning electron microscopy (SEM) analysis on lyophilized hydrogels. The bare Chit/Ag hydrogel displayed a uniform, interconnected macroporous structure resembling an “open-cell” architecture, characterized by irregular large pores with an average equivalent circular diameter (ECD) of 41.3 ±  31.9 μm (Figure 2a). However, when PEDOT:PSS was incorporated into the hydrogel matrix, a more close and entangled microstructure was observed while preserving high porosity and porous interconnectivity (Figure 2b–e). No significant changes in the porous structure were observed as the PEDOT:PSS content increased in the hydrogels. Thus, estimated ECDs sizes were 44.6  ±  38.3, 46.0  ±  33.3, 49.2  ±  38.1, and 52.7  ±  43.4 μm for the Chit/Ag/PEDOT:PSS(5), Chit/Ag/PEDOT:PSS(10), Chit/Ag/PEDOT:PSS(20), and Chit/Ag/PEDOT:PSS(30) hydrogels, respectively. Pore size in agarose-based hydrogels is known to depend largely on the cooling rate during gelation, with faster cooling leading to smaller pores and slower cooling resulting in larger pores.^36,37^ In our process, the hydrogel solutions were allowed to cool slowly, yielding large pores, in agreement with the results found in the literature (additional pore metrics are reported in Table S2, Supporting Information).

**Figure 2:**
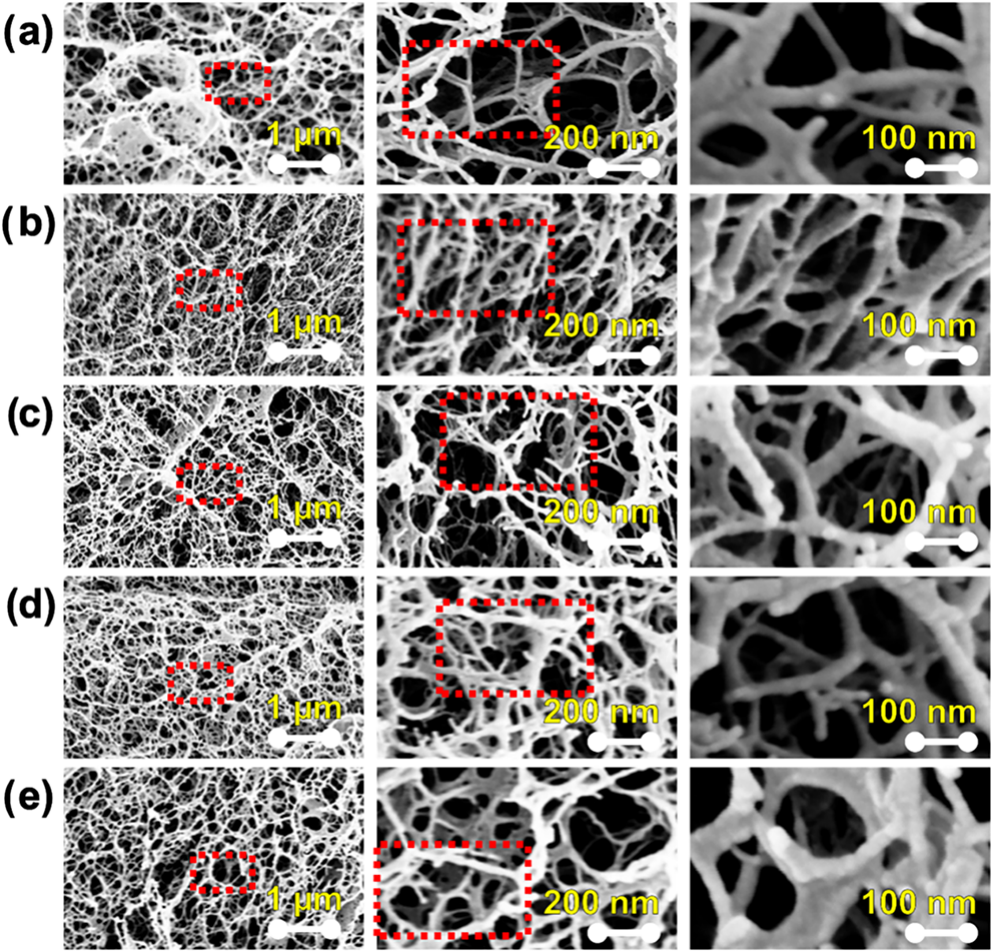
SEM images of chitosan-agarose films with different contents of PEDOT:PSS (a) 0 wt %, (b) 5 wt %, (c) 10 wt %, (d) 20 wt %, and (e) 30 wt %. The images in the same row correspond to different magnifications. Red rectangles show the regions where the high-magnification images were taken.

FTIR spectroscopy was conducted to investigate the chemical structure of the Chit/Ag hydrogel films and the effect of the presence of the conducting polymer on the Chit/Ag/PEDOT:PSS hydrogels (Figure 3). Chitosan and agarose are structurally similar polysaccharides, with the main distinction being that chitosan contains an amino (−NH_2_) group absent in agarose. In the FTIR spectrum of Chit/Ag hydrogel, a broad absorption band between 3400–3000 cm^–1^ is attributed to O–H and N–H stretching vibrations from hydroxyl (−OH) and amino (−NH_2_) groups present in both biopolymers.^38–41^ Additionally, a broad peak observed in the 2950–2825 cm^–1^ region correspond to methyl (−CH_3_) and methylene (−CH_2_) groups. Additionally, the peak at around 1650 cm^–1^ corresponds to the amide I band (C=O stretching), while the band near 1550 cm^–1^ can be assigned to amide II (N–H bending).^41^ Although chitosan typically exhibits a C–N stretching band around 2780 cm^–1^, this feature is not present in the spectra under study. These findings are consistent with previously published data.^31^ Upon incorporating PEDOT:PSS into the hydrogel matrix, the FTIR spectra of the composite films showed distinct changes, especially with increasing PEDOT concentrations. A new peak appeared around 1520–1550 cm^–1^, characteristic of the C=C stretching vibrations from the thiophene rings of PEDOT, confirming its successful incorporation into the hydrogel matrix.^42,43^ Furthermore, the peak at approximately 1250 cm^–1^, associated with the C–O–C stretching of the ethylenedioxy group in PEDOT is also noticeable. Interestingly, the broad O–H stretching band (3200–3400 cm^–1^) slightly shifted and decreased in intensity with higher PEDOT content, suggesting interactions between the hydroxyl groups of agarose/chitosan and the sulfonic acid groups in PSS.

**Figure 3:**
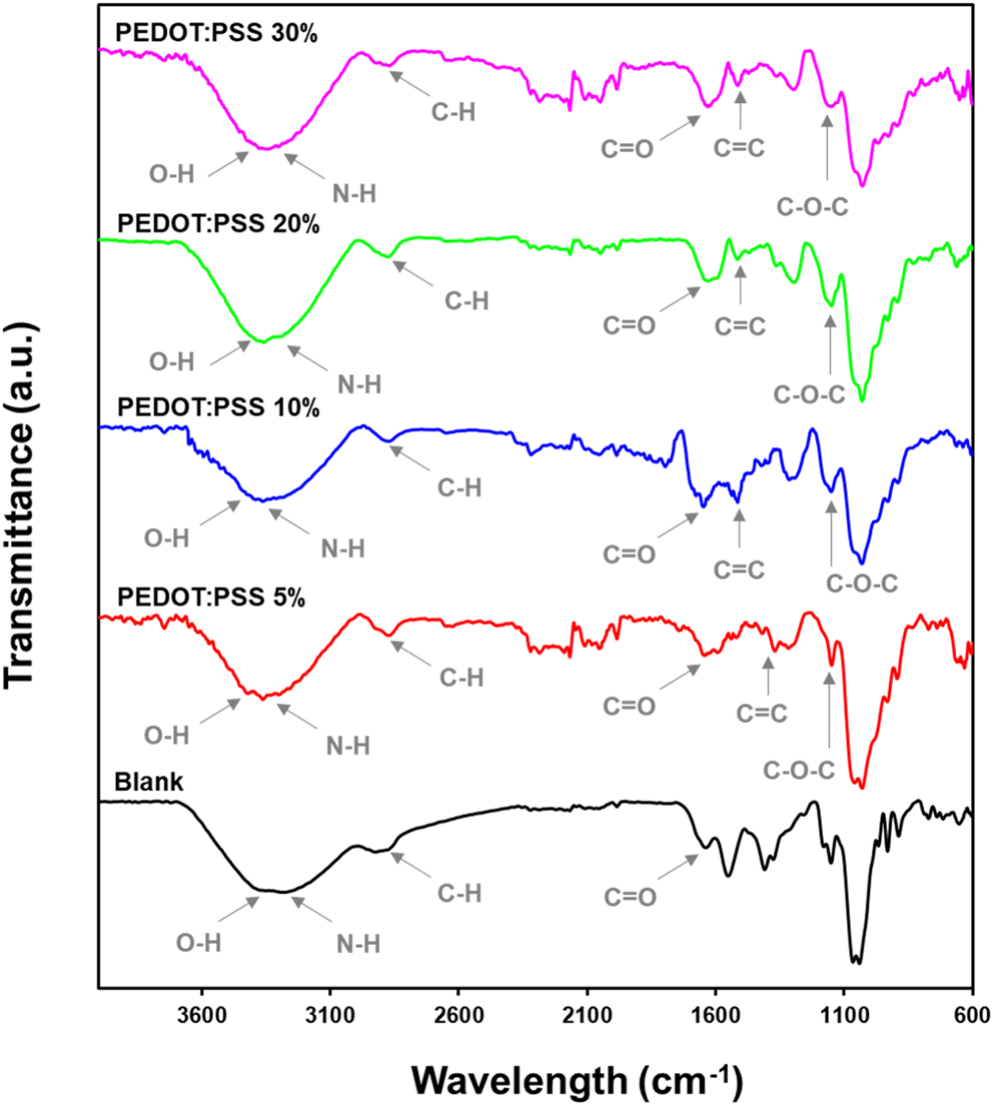
FTIR spectra of the Chitosan/Ag hydrogels with different content of PEDOT:PSS: 0, 5, 10, 20, and 30 wt %.

### 3.3 Swelling Behavior and Biodegradation

The water uptake capacity of the Chit/Ag/PEDOT:PSS hydrogels was evaluated by measuring their swelling ratios (SR), calculated as described in eq 1 (see Section 2). Hydrogels were immersed in distilled water at room temperature, and their weights were recorded at the initial time point and after 24 h of immersion. The resulting SR values are shown in Figure 4a. While bare Chit/Ag hydrogels exhibited the highest SR values (3393%), Chit/Ag/PEDOT:PSS hydrogels showed significantly lower SR values, in the range 747–1357%. Moreover, as the PEDOT:PSS content increased in the hydrogel, a gradual decrease in SR was observed. The fact of not having significant differences between hydrogels pore size with varying PEDOT:PSS content (as observed by SEM) suggests that changes in swelling behavior cannot be attributed to variations in porosity. Instead, other factors may be responsible for the observed trends, such as the influence of PEDOT:PSS on the polymer network structure. Thus, the reduction in SR with PEDOT:PSS content can be attributed to two main effects: (i) The incorporation of PEDOT.PSS leads to a denser or more constrained network that limits water uptake, and (ii) the intrinsic hydrophobicity and reduced flexibility of PEDOT:PSS may reduce the overall hydrogel hydrophilicity and flexibility, which therefore hinder hydrogel’s ability to absorb and retain water. Notably, despite the decrease in SR with higher PEDOT:PSS loading, all formulations maintained substantial swelling capacities, which is essential for ensuring intimate contact with biological tissues and effective temperature sensing performance in hydrated environments. These results are consistent with previous reports on composite hydrogels incorporating conductive polymers. For example, agarose/polyacrylamide (PAM)/poly­(vinyl alcohol) (PVA)/PEDOT:PSS hydrogels reported by Azar et al. exhibited similar trends, with SR values decreasing from ∼200 to ∼150% as PEDOT:PSS content increased.^44^ Likewise, PVA/PEDOT:PSS conductive hydrogels have shown lower SR, from 34 to 17%, due to increased network density.^45^ Overall, the swelling behavior observed here confirms the good water uptake ability of the Chit/Ag/PEDOT:PSS hydrogels, while also highlighting the tunability of this property via conductive polymer content.

**Figure 4:**
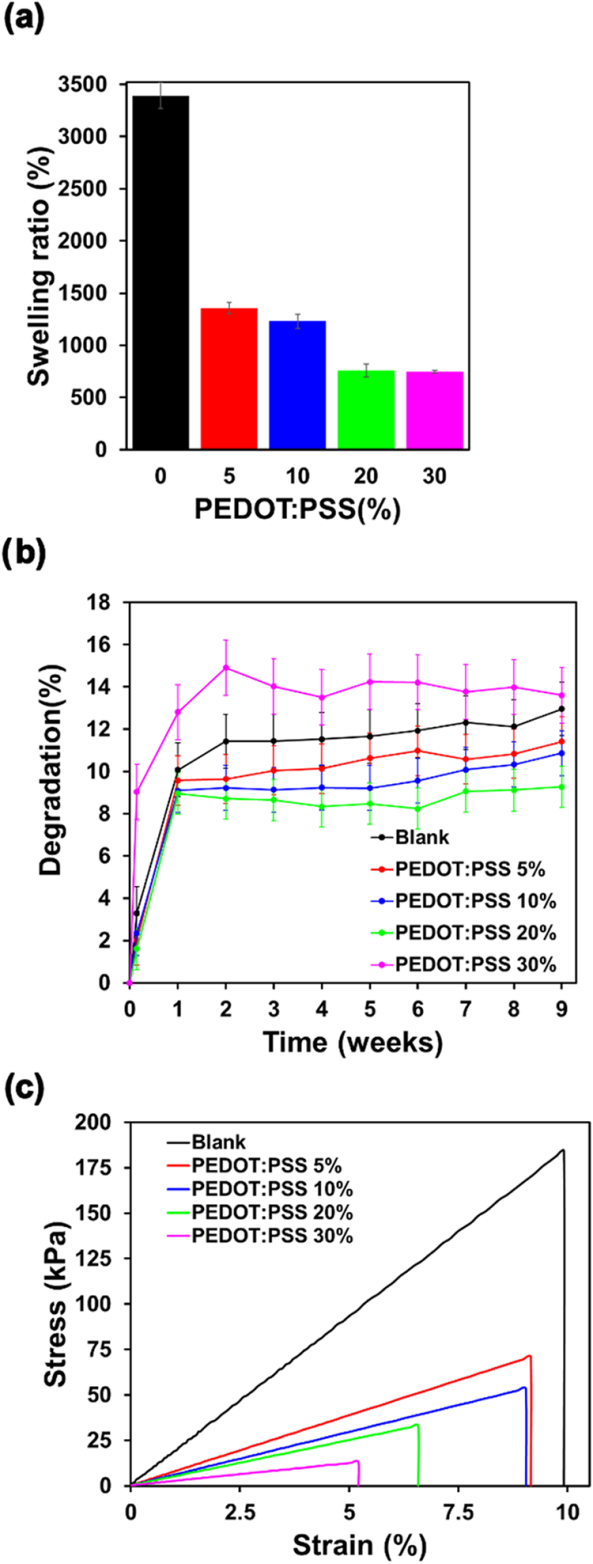
(a) Swelling ratio, (b) biodegradation rate, (c) tensile stress–strain curves of chitosan-agarose films with different PEDOT:PSS contents: 0, 5, 10, 20, and 30 wt %.

Biodegradation assays were conducted to evaluate the structural integrity and mass loss of the hydrogels in PBS during a 9-week immersion period (Figure 4b). All hydrogel formulations exhibited an initial weight loss during the first week, likely corresponding to the release of loosely bound components or surface erosion. After this initial phase, degradation slowed substantially, with only minor changes observed up to week 9, indicating good structural integrity over time. On the other hand, the results revealed concentration-dependent trends that aligned with previous expectations, but also presented notable exceptions (Figure 4b). Hydrogels containing 5, 10, and 20 wt % PEDOT:PSS showed reduced degradation compared to the blank Chit/Ag hydrogel, indicating that the incorporation of PEDOT:PSS contributes to improved hydrolytic stability of the network. This stabilizing effect is likely due to increased intermolecular interactions and partial shielding of the biopolymer matrix from water-mediated hydrolysis. These results are also in agreement with the SR values previously shown. Therefore, the lower the SR, the lower the absorption of PBS and the lower the degradation. Interestingly, the hydrogel with the highest PEDOT:PSS content (30 wt %) exhibited the highest degradation rate, surpassing even the blank formulation after the first week and continuing to degrade at a relatively elevated level throughout the study. This unexpected behavior suggests a nonmonotonic relationship between PEDOT:PSS content and matrix stability. While moderate levels of PEDOT:PSS reduces hydrogel degradation due to lower water absorption, an excess may lead to microstructural heterogeneity, reduced cross-linking efficiency, or even phase separation, ultimately weakening the integrity of the polymer network and increasing its susceptibility to degradation. These findings point to the existence of an optimal PEDOT:PSS concentration window that balances mechanical properties, swelling behavior and biodegradation. Beyond this threshold, the beneficial effects of PEDOT:PSS are diminished or reversed, likely due to the massive disruption of the chitosan–agarose matrix. This complex behavior has been observed in other composite hydrogels, where excessive filler content can induce structural defects or reduce cohesive interactions.^17^

### 3.4 Mechanical Properties

At the macroscopic level, all Chit/Ag/PEDOT:PSS hydrogels displayed good flexibility and mechanical integrity, facilitating easy handling and manipulation. The exception was the hydrogel with the highest PEDOT:PSS content (30%), which was brittle and prone to breakage during handling. The overall mechanical stability of the hydrogels can be attributed to multiple synergistic factors: (i) thermal gelation and cross-linking of agarose, (ii) pH-induced gelation of chitosan, (iii) van der Waals interactions between the polymer chains, and (iv) electrostatic interactions between the biopolymers and the PEDOT:PSS. To quantitatively evaluate mechanical performance, uniaxial tensile tests were conducted on hydrated hydrogels. The resulting stress–strain curves are shown in Figure 4c, while Table 1 summarizes key mechanical parameters, including Young’s modulus (*E*), tensile strength, and elongation at break for each hydrogel. The data show a clear trend: the incorporation of PEDOT:PSS leads to a progressive reduction in all measured mechanical properties. Hydrogels without PEDOT:PSS exhibited the highest stiffness, strength, and ductility. As PEDOT:PSS content increased, these values decreased consistently. This suggests that higher concentrations of PEDOT:PSS interfere with polymer chain entanglement or reduce cross-linking density, resulting in a softer and mechanically weaker network. Finally, to assess the suitability of these hydrogels for skin-related applications, the mechanical performance was compared with literature-reported values for human skin. All hydrogel formulations exhibited Young’s modulus values within the physiological range for human skin tissue (0.008–4 MPa), depending on measurement technique and anatomical location.^46–48^ The tensile strength of the hydrogels (0.01–0.18 MPa) and the elongation at break (5.2–9.9%) were slightly lower than the reported ranges for excised human skin (Tensile strength: 1–32 MPa, Ductility: 35–115%).^46–48^ Despite the lower tensile strength and ductility, the mechanical properties of these hydrogels are generally compatible with the mechanical requirements of soft tissue interfaces, particularly for applications such as flexible biosensors, wound healing patches, and transdermal drug delivery systems. Their tunable stiffness and mechanical robustness, combined with their conductivity and recyclability, make them promising candidates for skin-contact bioelectronic devices.

**Table 1:** Young’s Modulus, Tensile Strength, and Elongation at Break for All Fabricated Hydrogels^a^

hydrogel	Young modulus (MPa)	tensile strength (MPa)	elongation at break (%)
Chit/Ag	1.73 ± 0.22	0.18 ± 0.01	9.92 ± 1.14
Chit/Ag/PEDOT:PSS(5)	0.71 ± 0.15	0.07 ± 0.02	9.18 ± 1.85
Chit/Ag/PEDOT:PSS(10)	0.54 ± 0.09	0.05 ± 0.03	9.11 ± 1.47
Chit/Ag/PEDOT:PSS(20)	0.44 ± 0.16	0.03 ± 0.02	6.61 ± 1.32
Chit/Ag/PEDOT:PSS(30)	0.22 ± 0.04	0.02 ± 0.01	5.18 ± 1.16

a Results of the experiments are presented as mean ± standard deviation.

### 3.5 Electrochemical Properties

The electrochemical performance of the hydrogels was evaluated using cyclic voltammetry (CV) and electrochemical impedance spectroscopy (EIS) in three independent measurements, Figure 5 shows representative curves. The CV curves in Figure 5a correspond to hydrogels with varying PEDOT:PSS content. All samples exhibit nonrectangular yet symmetric voltammograms without discernible redox peaks. The nonrectangular shape indicates a deviation from ideal capacitive behavior, suggesting that the electrochemical response does not follow a purely electrical double-layer capacitance, which is often observed in PEDOT-based systems.^27^ The observed symmetry in the CV profiles reflects the good reversibility of the ion adsorption/desorption processes during potential cycling. The absence of redox peaks confirms the pseudocapacitive nature of the materials, consistent with the behavior of PEDOT:PSS. Furthermore, a slight distortion from ideal capacitive response—where ideal curves would be nearly rectangular—is observed at a scan rate of 100 mV s^–1^. This deviation is attributed to kinetic limitations associated with ion diffusion at higher scan rates. Importantly, the current density and the area enclosed by the CV curves (proportional to capacitance) increase progressively with PEDOT:PSS content. Specifically, compared to the bare Chit/Ag hydrogel, the CV area increases by approximately 110%, 171%, 207%, and 210% for hydrogels containing 5, 10, 20, and 30 wt.% PEDOT:PSS, respectively. This enhancement in capacitive response is ascribed to the improved electrical conductivity and intrinsic pseudocapacitance of PEDOT:PSS, which facilitate greater charge transport and storage within the hydrogel matrix. The corresponding values of areal capacitance are 189.5 ± 0.5, 214.3 ± 0.1, 334.9 ± 0.3, 404.9 ± 0.3, and 413.9 ± 0.7 mF cm^–2^.

**Figure 5:**
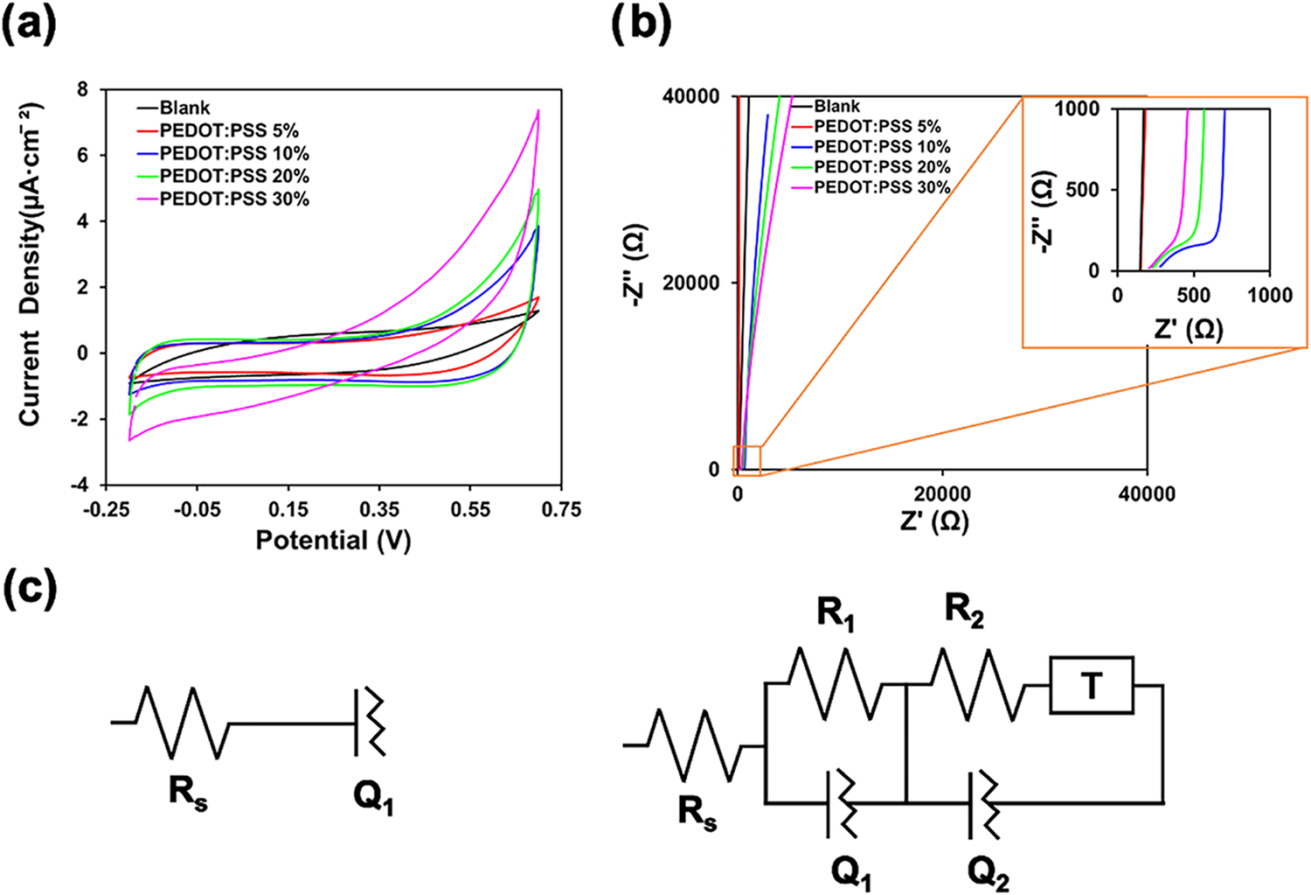
(a) Cyclic voltammograms recorded at 100 mV/s, (b) Nyquist plot of Chit/Ag, Chit/Ag/PEDOT:PSS(5), Chit/Ag/PEDOT:PSS(10), Chit/Ag/PEDOT:PSS(20) and Chit/Ag/PEDOT:PSS(30) hydrogels, (c) the equivalent electrical circuits.

The EIS results are presented in the Nyquist plots (Figure 5b) and interpreted using equivalent electrical circuit models (Figure 5c). Figure S1 (Supporting Information) presents the individual Nyquist plots for each hydrogel, with the low- and high-frequency regions clearly indicated for better interpretation. Two different circuit configurations were employed, depending on the PEDOT:PSS content in the hydrogels. For the Chit/Ag (blank) and Chit/Ag/PEDOT:PSS(5) hydrogels, a simplified equivalent circuit consisting of a series resistance (R_s_) and a constant phase element (*Q*_1_) was sufficient to describe the system’s response (Figure 5c, left). In this model, *R*_s_ corresponds to the bulk resistance of the electrolyte and electrode contacts, while *Q*_1_ accounts for nonideal capacitive behavior at the hydrogel/electrode interface. In contrast, for hydrogels with higher PEDOT:PSS content, a more complex equivalent circuit was necessary to accurately fit the impedance data (Figure 5c, right). This model includes two *R*–*Q* branches and a finite-length Warburg-type transmission line element (*T*), capturing the increased electrochemical activity and ion transport within the hydrogel matrix. The *R*_p1_–*Q*_1_ branch represents interfacial charge transfer resistance and associated pseudocapacitive processes, while the *R*_p2_–*Q*_*2*_–*T* branch reflects slower ion diffusion and charge storage within the bulk of the hydrogel, influenced by the presence of PEDOT conductive domains.^49–51^ The evolution of the circuit parameters with PEDOT:PSS content is summarized in Table 2. The solution resistance *R*_s_ remains nearly constant (∼146–152 Ω), indicating that the bulk ionic conductivity of the system (primarily governed by PBS and the substrate configuration) is not significantly affected by PEDOT:PSS concentration. However, the values of *Q*_1_ and *Q*_2_ increase with PEDOT:PSS loading, signifying enhanced charge storage capability. This trend aligns with the CV data and is attributed to improved electronic conductivity and higher interfacial area provided by the conducting polymer. The exponents *n*_1_ and *n*_2_, which characterize the deviation of *Q*_1_ and *Q*_2_ from ideal capacitive behavior, approach unity with increasing PEDOT:PSS concentration. This suggests a more homogeneous and capacitive interface, consistent with the formation of well-distributed conductive networks within the hydrogel.^49,50^ The charge transfer resistances *R*_p1_ and *R*_p2_ decrease systematically as PEDOT:PSS increases, further confirming enhanced electron and ion transport pathways. The transmission line element *T*, which models diffusion-limited processes, becomes significant at 10–30% PEDOT:PSS content, reaching its maximum value at 10% and decreasing slightly thereafter. This suggests that moderate PEDOT:PSS incorporation optimizes ion transport by creating interconnected pathways, whereas excessive loading may introduce morphological constraints that hinder diffusion.^51^ The parameter *B*, associated with diffusion length, remains relatively constant across all hydrogels, indicating that the structural aspects of the matrix governing this parameter were not significantly affected by PEDOT:PSS concentration. The fitting quality of the equivalent circuits was evaluated through the chi-squared (χ^2^) parameter, which was found to be on the order of 10^–3^ for all samples. This low χ^2^ value indicates an excellent agreement between the experimental impedance data and the proposed equivalent circuit models. In summary, the impedance analysis confirms that increasing PEDOT:PSS content enhances the electronic and capacitive properties of the hydrogels by lowering charge transfer resistance and increasing interfacial capacitance. However, at higher loadings, morphological effects may begin to limit ion transport, suggesting an optimal PEDOT:PSS concentration for maximizing electrochemical performance.

**Table 2:** Numerical Results from EIS Data Fitting for All the Elements of the Electric Equivalent Circuits

element	blank	PEDOT 5%	PEDOT 10%	PEDOT 20%	PEDOT 30%
*R*_s_ (Ω)	145.95 ± 11.07	151.95 ± 12.02	149.98 ± 11.14	147.56 ± 11.89	146.52 ± 13.12
*R*_p1_ (Ω)	**---**	**---**	411.98 ± 14.04	307.19 ± 10.93	211.37 ± 17.11
*Q*_1_ (μS·s^*n*1^)	27.25 ± 0.79	29.98 ± 0.99	18.32 ± 1.02	20.08 ± 0.88	27.39 ± 0.77
*n*_1_	0.98 ± 0.01	0.99 ± 0.02	0.70 ± 0.01	0.78 ± 0.02	0.81 ± 0.01
*R*_p2_ (Ω)	**---**	**---**	128.49 ± 11.13	89.98 ± 12.14	64.84 ± 9.37
*Q*_2_ (μS·s^*n*2^)	**---**	**---**	2.76 ± 0.86	4.10 ± 0.23	5.18 ± 1.20
*n*_2_	**---**	**---**	0.51 ± 0.02	0.56 ± 0.01	0.57 ± 0.01
*T* (μS·s^0.5^)	**---**	**---**	795.98 ± 12.33	642.46 ± 15.76	542.90 ± 19.23
*B* (s^0.5^)	**---**	**---**	0.05 ± 0.01	0.05 ± 0.01	0.04 ± 0.01
χ^2^	4.9 × 10^–3^ ± 2.2 × 10^–3^	7.1 × 10^–3^ ± 2.5 × 10^–3^	6.8 × 10^–3^ ± 3.0 × 10^–3^	7.0 × 10^–3^ ± 2.2 × 10^–3^	7.9 × 10^–3^ 1.6 ± × 10^–3^

### 3.6 Proof of Concept: Temperature Sensing

Finally, the fabricated Chit/Ag/PEDOT:PSS hydrogels were evaluated as temperature sensors since PEDOT:PSS is a thermoresistor material, allowing sensing the variation of resistance with temperature.^52,53^ To do so, hydrogels were cut into 0.5 cm × 4 cm stripes, contacted by copper stripes and immersed in a water bath. The temperature of the water bath was changed from room temperature (∼23 °C) to 50 °C and the normalized resistance (Δ*R*/*R*_0_) evaluated in this range. As it can be observed in Figure 6, the normalized resistance decreases as the temperature increases for all PEDOT:PSS-containing hydrogels, which is due to the enhanced electrical conductivity of PEDOT. As a semiconducting material, PEDOT exhibits thermally activated charge transport; higher temperatures promote both the mobility and the generation of charge carriers, thereby reducing electrical resistance. Additionally, the slope of this representation, which is the TCR (see eq 4, Section 2), defines the sensitivity of the hydrogel. The slope becomes steeper as the PEDOT:PSS content increases in the hydrogel, therefore, the TCR and sensitivity of the hydrogels increases from −0.1 to −1.5% °C^–1^ in the order: Chit/Ag < Chit/Ag/PEDOT(5) < Chit/Ag/PEDOT(10) < Chit/Ag/PEDOT(20) < Chit/Ag/PEDOT(30), results summarized in Figure 6b. As the PEDOT:PSS content increases, its conductive network within the hydrogel becomes more interconnected, facilitating electron transport and amplifying the thermal response. The steeper slope observed with higher PEDOT content may result from a stronger dependence of resistance on temperature due to the dominance of PEDOT’s conductive mechanism over the insulating properties of chitosan and agarose. The highest sensitivities are obtained for the Chit/Ag/PEDOT(20) and Chit/Ag/PEDOT(30) hydrogels, which are higher than the values reported for other hydrogels based on conducting polymers like PEDOT:PSS or PANI (see Table 3). For example, PEDOT:PSS hydrogels incorporating different carbon type nanomaterials (graphene, carbon nanoparticles, carbon nanotubes) show TCR values in the range from −0.06 to −0.61% °C^–1^,^53–55^ which are lower than the values reported in the work. Thus, we would like to highlight that the temperature sensitivity of the PEDOT:PSS-based hydrogels developed is superior to many existing hydrogel sensors.

**Figure 6:**
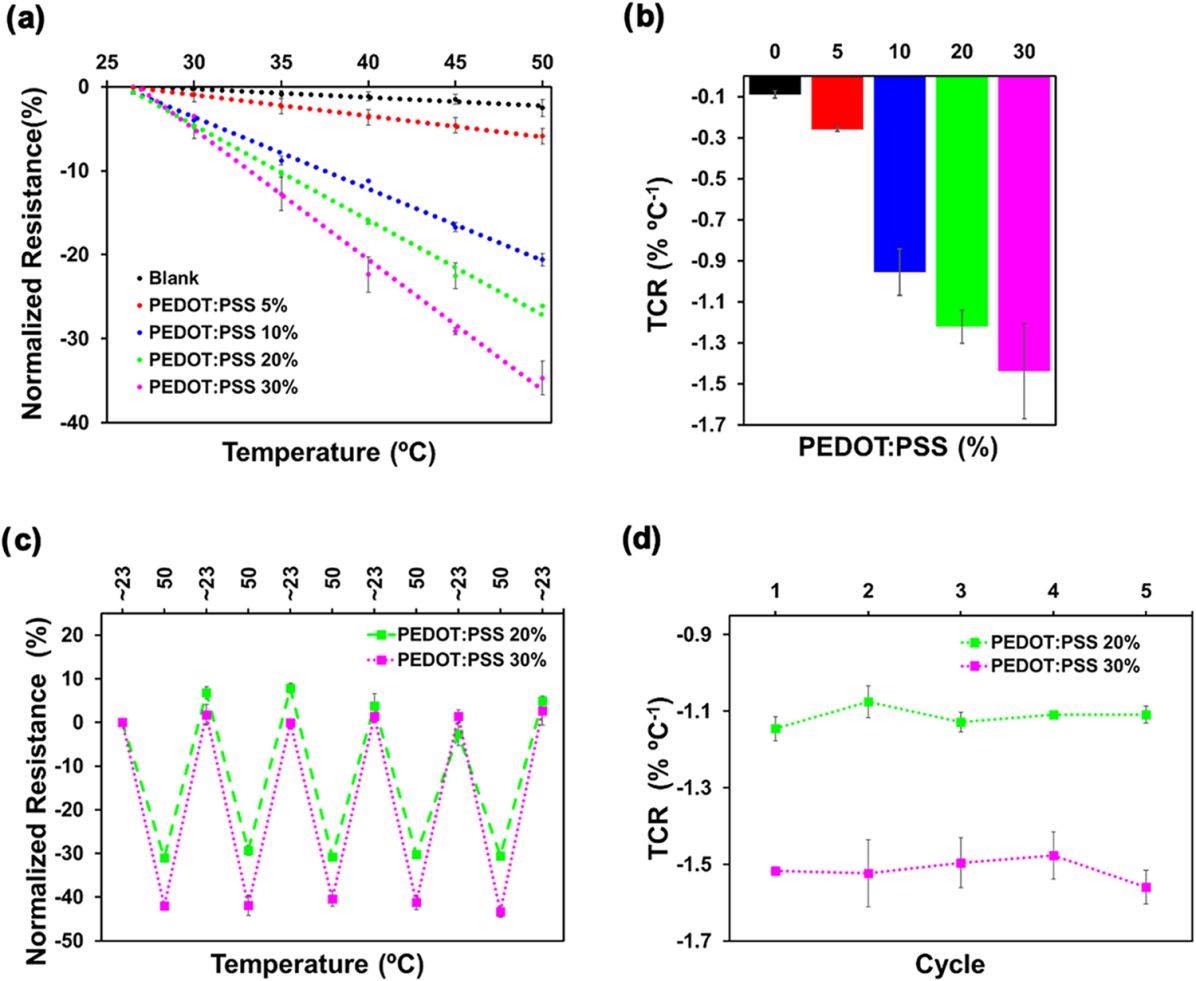
(a) Normalized resistance as a function of temperature, (b) TCR of chitosan-agarose hydrogels with different PEDOT:PSS contents: 0, 5, 10, 20, and 30 wt %, (c) normalized resistance through heating–cooling cycles between *T*_0_ ∼ 23 °C and *T*_1_ = 50 °C of Chit/Ag/PEDOT:PSS(20) and Chit/Ag/PEDOT:PSS(30) hydrogels, (d) TCR after each heating–cooling cycle.

**Table 3:** Comparison of TCR Values for Similar Conductive Hydrogels Reported in the Literature^a^

hydrogel	TCR (% °C^–1^)	refs
PEDOT:PSS/graphene	–0.06	^54^
PEDOT:PSS/CNP	–0.25	^55^
PEDOT:PSS/CNT	–0.61	^53^
PANI electropolimerized on PET	–1.0	^56^
alginate/PEDOT/CNP/MnO_2_	–1.05	^57^
alginate/PEDOT/Fe_3_O_4_	–1.35	^58^

a Acronyms: CNP: Carbon nanoparticles, CNT: Carbon nanotubes, PANI: Polyaniline, PET: Poly­(ethylene terephthalate).

On the other hand, a practically negligible hysteresis was observed after decreasing from 60 °C to room temperature. The heating–cooling cycle was repeated several times showing practically no differences in the normalized resistances and corresponding TCR values (Figure 6c,d), proving the excellent reproducibility, and therefore performance, of the hydrogel as temperature sensor. This stability in the measurements suggests that the structure of the hydrogel and the conductive network formed by PEDOT:PSS are not significantly altered by thermal cycling within the tested temperature range.

To further evaluate the time-dependent response of the Chit/Ag/PEDOT:PSS(20) hydrogel sensor, two complementary experiments were conducted. First, the response and recovery times were determined by analyzing the resistance variation during periodic contact and isolation from a temperature-controlled heat source. The sensor was cycled between room temperature (23 °C) and 37 °C to simulate physiological conditions. As shown in Figure S2, the resistance decreased reproducibly upon heating and recovered upon cooling. The response time (defined as the time to reach 90% of the steady-state signal upon heating) was 12.1 s, while the recovery time (cooling back to 90% of baseline) was 15.2 s. These values correspond to an average response rate of 0.86 ± 0.21 s·°C^–1^ and recovery rate of 1.09 ± 0.44 s·°C^–1^, indicating fast and reversible temperature sensing. No baseline drift or hysteresis was observed after repeated heating/cooling cycles, confirming good signal stability. And second, we evaluated the operation of the hydrogel in a humid biological environment. Thus, the Chit/Ag/PEDOT:PSS(20) film was immersed in cell culture medium inside a humidified incubator (37 °C, 95% RH). The resistance was continuously recorded while the medium equilibrated from room temperature (23 °C) to 37 °C, and again during spontaneous cooling after removal from the incubator. The sensor demonstrated prompt and stable resistance changes in both directions (Figure S3), maintaining signal reproducibility without delay or overshoot. Moreover, the fact that hydrogels display a stable signal after a few hours immersed in PBS signal indicates that biodegradation (which is very low over a 9 week period) does not alter the electrical performance.

Finally, the electrical performance of the Chit/Ag/PEDOT:PSS(20) hydrogel film under compressive stress was evaluated. To do so, known weights were placed on the Chit/Ag/PEDOT:PSS(20) film, producing pressures from 0.15 to 0.61 kPa. The relative normalized resistance decreased slightly from −0.90% to −1.16%. This confirms that within the pressure regime tested, the electrical signal was nearly independent of normal stress, indicating negligible cross-sensitivity between temperature and pressure in this range (Figure S4).

### 3.7 Reusability

Reusability remains a major challenge in the development of soft electronic devices based on conductive hydrogels. Ideally, hydrogel-based devices that become nonfunctional due to mechanical damage or dehydration could be easily reprocessed and reused for the same purpose. Achieving true reusability is essential to address the growing problem of electronic waste, reduce the demand for raw materials, and lower production and consumer costs. While recent studies have explored “reusable” hydrogels, they typically refer to either regeneration—restoring lost function through simple treatments like rehydration—or recycling, where materials are dissolved and repurposed, often with reduced performance. For instance, Liu et al. restored swelling in polyacrylamide–alginate hydrogels via water immersion, maintaining functionality without altering the device structure.^59^ In contrast, Pereira et al. recycled cellulose-based hydrogels by dissolving and reforming them, but the resulting materials showed inferior electronic performance, limiting their reuse in the original application.^60^ In this work, we establish a clear and practical definition of reusability in hydrogel-based electronics: the ability to disassemble, reprocess, and reuse the hydrogel in its original function—without significant loss of performance. This distinction sets our approach apart from conventional regeneration or recycling and represents a meaningful step toward sustainable, circular-use soft electronic devices.

We followed a simple procedure to disassemble the hydrogel and reprocess it. Thus, the hydrogel was mixed with a small volume of water, heated up to 85 °C to enhance agarose de-cross-linking, the mixture homogenized, poured in a Petri dish and cooled down to room temperature for 4 h. After that, temperature-sensing tests were performed on the recycled samples. This process was performed for the Chit/Ag/PEDOT(20) and Chit/Ag/PEDOT(30) hydrogels. While Chit/Ag/PEDOT(20) hydrogel was successfully recycled 3 times without significant loss of functionality, Chit/Ag/PEDOT(30) failed to rehomogenize and form a gel the first time. The inability of the 30% PEDOT:PSS sample to rehomogenize suggests that an excessively high concentration of PEDOT:PSS disrupts the gelation process, potentially due to phase separation or an imbalance in the chitosan-agarose-PEDOT:PSS interaction. This highlights a limit to the amount of PEDOT:PSS that can be incorporated into the hydrogel while maintaining its reusability. Figure 7 shows the variation of normalized resistance for different heating–cooling cycles (Figure 7a) and the TCR values (Figure 7b) for the as-prepared hydrogel and the first and second time reused Chit/Ag/PEDOT(20) hydrogels. No significant differences were detected among the as-prepared and reused hydrogels, suggesting that the hydrogel performance remains stable upon reuse. The successful recycling of the Chit/Ag/PEDOT(20) hydrogel demonstrates the resilience and reproducibility of the material under appropriate conditions. This suggests that the hydrogel structure and conductive network can be effectively reformed after disassembly, emphasizing the material’s potential for sustainable applications. The balance between conductivity and structural integrity achieved with Chit/Ag/PEDOT(20) hydrogel makes it an optimal composition for both functional performance and reusability.

**Figure 7:**
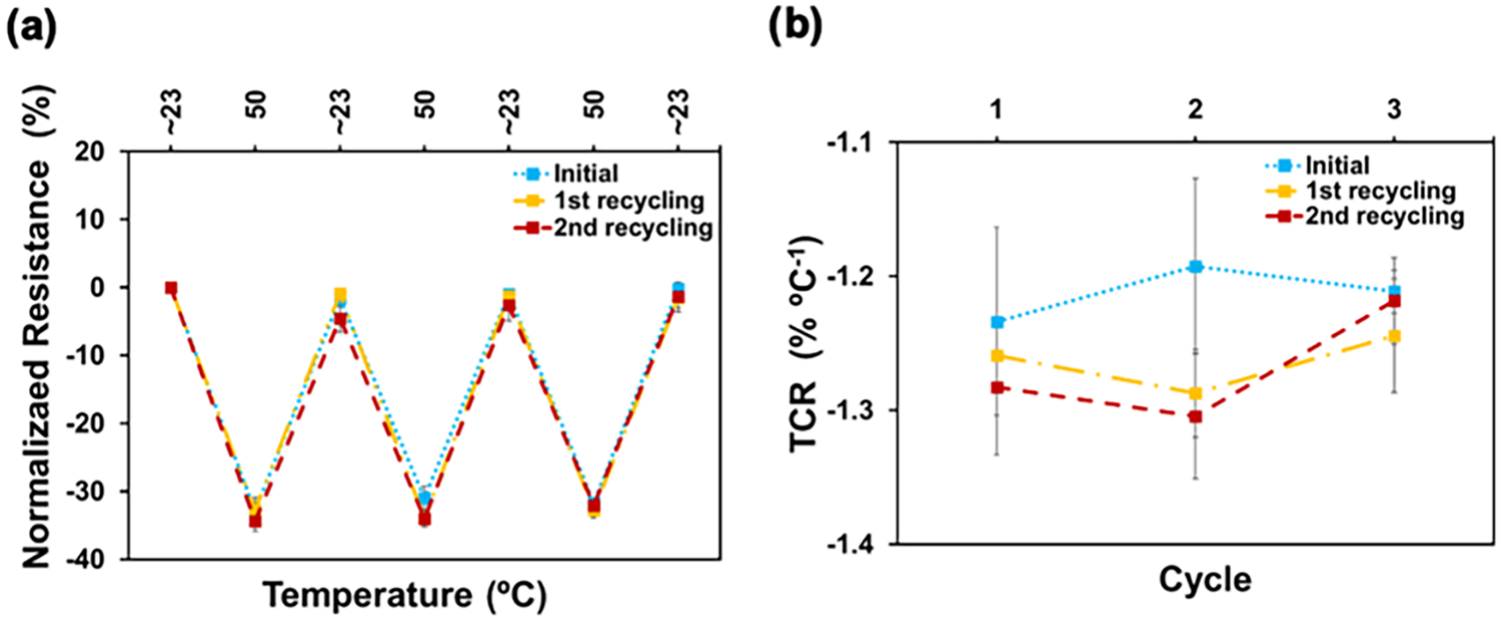
(a) Normalized resistance and (b) TCR through heating–cooling cycles between *T*_0_ ∼ 23 °C and *T*_1_ = 50 °C of as-prepared Chit/Ag/PEDOT(20) hydrogels and after two recycling processes.

Beyond their biodegradability, the environmental relevance of these hydrogels lies in their demonstrated recyclability and potential for circular reuse. The reprocessing of Chit/Ag/PEDOT(20) hydrogels can be achieved simply by heating the material in water (85–95 °C) without the need for solvents or additional reagents, consuming less energy than the required for new fabrication. This low-energy process allows the material to be remolded and reused with negligible loss of performance, as demonstrated in the temperature-sensing tests after multiple cycles. Moreover, material costs are practically null per reuse cycle since the biopolymer components can be recovered and reprocessed with minimal degradation. Importantly, the entire procedure generates no toxic or persistent waste, and any residual material remains biodegradable. These features highlight that the recyclability of the hydrogels provides both environmental and economic advantages, complementing their sustainable fabrication and supporting their integration within a circular materials framework for soft bioelectronics. Despite the advantages of the developed chitosan–agarose–PEDOT:PSS hydrogels, further improvements are needed to enhance their ductility and stretchability while preserving their electrochemical performance. Future efforts will focus on optimizing the polymer network architecture and incorporating flexible components to achieve greater mechanical compliance without compromising conductivity and reusability, thereby extending their applicability in soft and deformable bioelectronic systems.

## 4 Conclusions

In summary, we have developed a dual chitosan–agarose hydrogel integrated with PEDOT:PSS as sustainable, recyclable, soft, and thermoresponsive materials for skin-integrated temperature sensing. These hydrogels demonstrate high swelling capacities, mechanical properties compatible with human skin, and enhanced electrochemical performance, achieving temperature sensitivities superior to many existing hydrogel-based sensors. The materials maintain stable performance under repeated thermal cycling and can be reprocessed and reused without significant loss of functionality, addressing sustainability challenges in soft electronics. The integration of conductivity, recyclability, and biofunctionality within a green and easy fabrication process positions these hydrogels as promising sustainable platforms for the development of next-generation wearable healthcare monitoring devices within a circular materials economy.

## Supplementary Material



## Data Availability

Data available on request from the authors.

## Notes

Additional experimental details (PEDOT:PSS dispersion volume), hydrogel’s pore metrics, Nyquists plots of hydrogels, response and recovery, continuous temperature monitoring and variation of normalized resistance vs pressure of the Chit/Ag/PEDOT:PSS(20) hydrogel as sensor (PDF)

## References

[1] Sharma S., Tiwari S. (2020). A review on biomacromolecular hydrogel classification
and its applications. Int. J. Biol. Macromol..

[2] Dragan E. S. (2014). Design
and applications of interpenetrating polymer network hydrogels. A
review. Chem. Eng. J..

[3] Zhang Y. S., Khademhosseini A. (2017). Advances in
engineering hydrogels. Science.

[4] Kuzina M. A., Kartsev D. D., Stratonovich A. V., Levkin P. A. (2024). Organogels versus
Hydrogels: Advantages, Challenges, and Applications. Adv. Funct. Mater..

[5] Dechiraju H., Jia M. P., Luo L., Rolandi M. (2021). On-Conducting Hydrogels
and Their Applications in Bioelectronics. Adv.
Sustainable Syst..

[6] Hao X. P., Li C. Y., Zhang C. W., Du M., Ying Z. M., Zheng Q., Wu Z. L. (2021). Self-Shaping Soft
Electronics Based
on Patterned Hydrogel with Stencil-Printed Liquid Metal. Adv. Funct. Mater..

[7] Ghanbari M., Salavati-Niasari M., Mohandes F. (2022). Nanocomposite scaffolds based on
gelatin and alginate reinforced by Zn2SiO4 with enhanced mechanical
and chemical properties for tissue engineering. Arabian J. Chem..

[8] García-Torres, J. ; Aleman, C. ; Gupta, R. K. Multifunctional Hydrogels: From Basic Concepts to Advanced Applications; CRC Press, Taylor & Francis Group: New York, 2024, 9781032373409.

[9] Karchoubi F., Ghotli R. A., Pahlevani H., Salehi M. B. (2024). New insights into
nanocomposite hydrogels; a review on recent advances in characteristics
and applications. Adv. Ind. Eng. Polym. Res..

[10] Ghanbari M., Salavati-Niasari M., Mohandes F. (2021). Thermosensitive alginate-galtin-nitrogen-doped
carbon dots scaffolds as potential injectable for cartilage tissue
engineering applications. RSC Adv..

[11] Yuk H., Lu B., Zhao X. (2019). Hydrogel bioelectronics. Chem.
Soc. Rev..

[12] Garcia-Torres, J. Hybrid Hydrogels with Stimuli-Responsive Properties to Electric and Magnetic Fields. In Hydrogels—From Tradition to Innovative Platforms with Multiple Applications; Popa, L. , Ed.; IntechOpen: London, 2022, 978-1-80355-583-6.

[13] Someya T., Bao Z., Malliaras G. G. (2016). The rise
of plastic bioelectronics. Nature.

[14] Lacour S. P., Courtine G., Guck J. (2016). Materials
and technologies for soft
implantable neuroprostheses. Nat. Rev. Mater..

[15] García-Torres J., Colombi S., Macor L. P., Aleman C. (2022). Multitasking smart
hydrogels based on the combination of alginate and poly­(3,4-ethylenedioxythiophene)
properties: A review. Int. J. Biol. Macromol..

[16] Alamdari S. G., Alibakhshi A., de la Guardia M., Baradaran R., Mohammadzadeh R., Amini M., Kesharwani P., Mokhtardadeh A., Oroojalian F., Sahebkar A. (2022). Conductive and Semiconductive
Nanocomposite-Based Hydrogels for Cardiac Tissue Engineering. Adv. Healthcare Mater..

[17] Gamboa J., Paulo-Mirasol S., Espona-Noguera A., Enshaei H., Ortiz S., Estrany F., Ginebra M.-P., Torras J. (2024). Biodegradable conducting
PVA-hydrogel based on carbon quantum dots: Study of the synergistic
effect of additives. J. Polym. Environ..

[18] Naranjo D., Paulo-Mirasol S., Lanzalaco S., Armelin E., Garcia-Torres J., Torras J. (2024). Thermosensitive Hydrogel PNIPAAm-Alg-PEDOT for Sustainable
and Efficient Water Purification Powered by Solar Energy. Adv. Sustainable Syst..

[19] Guo B., Glavas L., Albertsson A. C. (2013). Biodegradable and electrically conducting
polymers for biomedical applications. Prog.
Polym. Sci..

[20] Fu F., Wang J., Zeng H., Yu J. (2020). Functional conductive
hydrogels for bioelectronics. ACS Mater. Lett..

[21] Kayser L. V., Lipomi D. J. (2019). Stretchable Conductive
Polymers and Composites Based
on PEDOT and PEDOT:PSS. Adv. Mater..

[22] Babeli I., Puiggali-Jou A., Roa J. J., Ginebra M.-P., Garcia-Torres J., Aleman C. (2021). Hybrid conducting alginate-based hydrogel for hydrogen
peroxide detection from enzymatic oxidation of lactate. Int. J. Biol. Macromol..

[23] Guo M. L., Yang X., Yan J., An Z. J., Wang L., Wu Y. P., Zhao C. X., Xiang D., Li H., Li Z. Y., Zhou H. W. (2022). Anti-Freezing,
Conductive and Shape
Memory Ionic Glycerol-Hydrogels with Synchronous Sensing and Actuating
Properties for Soft Robotics. J. Mater. Chem.
A.

[24] Ferlauto L., D’Angelo A. N., Vagni P., Leccardi M. J. I. A., Mor F. M., Cuttaz E. A., Heuschkel M. O., Stoppini L., Ghezzi D. (2018). Development and Characterization
of PEDOT:PSS/Alginate Soft Microelectrodes for Application in Neuroprosthetics. Front. Neurosci..

[25] Wang Z., Wang T., Zhuang M., Xu H. (2019). Stretchable Polymer
Composite with a 3D Segregated Structure of PEDOT:PSS for Multifunctional
Touchless Sensing. ACS Appl. Mater. Interfaces.

[26] Banitaba S. N., Khademolqorani S., Jadhav V. V., Chamanehpour E., Mishra Y. K., Mostafavi E., Kaushik A. (2023). Recent progress of
bio-based smart wearable sensors for healthcare Applications. Mater. Today Electron..

[27] Babeli I., Ruano G., Casanovas J., Ginebra M.-P., Garcia-Torres J., Aleman C. (2020). Conductive, self-healable
and reusable poly­(3,4-ethylenedioxythiophene)-based
hydrogels for highly sensitive pressure arrays. J. Mater. Chem. C.

[28] Keene S. T., Guiskine V., Berggren M., Malliaras G. G., Tybrandt K., Zozoulenko I. (2022). Exploiting mixed conducting polymers
in organic and bioelectronic devices. Phys.
Chem. Chem. Phys..

[29] Torras J., Casanovas J., Alemán C. (2012). Reviewing extrapolation procedures
of the electronic properties on the π-conjugated polymer limit. J. Phys. Chem. A.

[30] Hu L., Chee P. L., Sugiarto S., Yu Y., Shi C., Yan R., Yao Z., Shi X., Zhi J., Kai D., Yu H.-D., Huang W. (2023). Hydrogel-Based Flexible Electronics. Adv. Mater..

[31] Viteri A., Espanol M., Ginebra M.-P., Garcia-Torres J. (2025). Tailoring
drug release from skin-like chitosan-agarose biopolymer hydrogels
containing Fe_3_O_4_ nanoparticles using magnetic
fields. Chem. Eng. J..

[32] Rinaudo M. (2006). Chitin and
chitosan: Properties and applications. Prog.
Polym. Sci..

[33] Beaumont M., Tran R., Vera G., Niedrist D., Rousset A., Pierre R., Shastri V. P., Forget A. (2021). Hydrogel-Forming Algae
Polysaccharides: From Seaweed to Biomedical Applications. Biomacromolecules.

[34] Annabi N., Shin S. R., Tamayol A., Miscuglio M., Bakooshli M. A., Assmann A., Mostafalu P., Sun J.-Y., Mithieux S., Cheung L., Tang X., Weiss A. S., Khademhosseini A. (2016). Highly Elastic and Conductive Human-Based
Protein Hybrid Hydrogels. Adv. Mater..

[35] Kougkolos G., Golzio M., Laudebat L., Valdez-Nava Z., Flahaut E. (2023). Hydrogels with electrically conductive
nanomaterials
for biomedical applications. J. Mater. Chem.
B.

[36] Jayawardena I., Turunen P., Garms B. C., Rowan A., Corrie S., Grøndahl L. (2023). Evaluation
of Techniques Used for Visualisation of
Hydrogel Morphology and Determination of Pore Size Distributions. Mater. Adv..

[37] Chen C., Li X., Zhao D., Li Y., Shi H., Ma G., Su Z. (2017). Precise Control of Agarose Media Pore Structure by Regulating Cooling
Rate. J. Sep. Sci..

[38] Resina L., Esteves T., Pérez-Rafael S., García J. I. H., Ferreira F. C., Tzanov T., Bonardd S., Díaz D. D., Pérez-Madrigal M. M., Alemán C. (2024). Dual Electro-/PH-Responsive
Nanoparticle/Hydrogel System for Controlled Delivery of Anticancer
Peptide. Biomater. Adv..

[39] Banach-Kopeć A., Mania S., Tylingo R., Wawrzynowicz A., Pawłowska M., Czerwiec K., Deptuła M., Pikuła M. (2024). Thermosensitive Composite Based on Agarose and Chitosan
Saturated with Carbon Dioxide, Preliminary Study of Requirements for
Production of New CSAG Bioink. Carbohydr. Polym..

[40] Su W., Chen J., Zhang Y., Luo X., Lin C., Li P. (2024). Chitosan/Agarose Hydrogel Dressing:
PH Response Real-Time Monitoring
and Chemo-/Photodynamic Therapy Synergistic Treatment of Infected
Wounds. Int. J. Biol. Macromol..

[41] Wang Y., Yang W., Zhu J., Chen Q., Li N. (2023). Agarose Hydrogel
Doped with Soluble Se-Chitosan for Se-Enriched Cultivation of Sprouts. Adv. Agrochem..

[42] Sakunpongpitiporn P., Phasuksom K., Paradee N., Sirivat A. (2019). Facile Synthesis of
Highly Conductive PEDOT:PSS: Via Surfactant Templates. RSC Adv..

[43] Pasha A., Roy A. S., Murugendrappa M. V., Al-Hartomy O. A., Khasim S. (2016). Conductivity and Dielectric Properties
of PEDOT-PSS
Doped DMSO Nano Composite Thin Films. J. Mater.
Sci.: Mater. Electron..

[44] Azar M. G., Dodda J. M., Bělský P., Šlouf M., Vavruňková V., Kadleca J., Remiš T. (2021). Though and
lexible conductive triple network hydrogels based on agarose/polyacrylamide/
polyvinyl alcohol and poly (3,4-ethylenedioxythiophene):polystyrene
sulfonate. Polym. Int..

[45] Zhang Y.-F., Guo M.-M., Zhang Y., Tang C. Y., Jiang C., Dong Y., Law W.-C., Du F.-P. (2020). Flexible, stretchable
and conductive PVA/PEDOT:PSS composite hydrogels prepared by SIPN
strategy. Polym. Test..

[46] Ottenio M., Tran D., Annaidh A. N., Gilchrist M. D., Bruyère K. (2015). Strain rate and anisotropy effects
on the tensile failure
characteristics of human skin. J. Mech. Behav.
Biomed. Mater..

[47] Kalra A., Lowe A., Al-Jumaily A. (2016). Mechanical
behaviour of skin: a review. J. Mater. Sci.
Eng..

[48] Annaidh, A. N. ; Ottenio, M. ; Bruyère, K. ; Destrade, M. ; Gilchrist, M. D. Mechanical Properties of Excised Human Skin. In 6th World Congress of Biomechanics (WCB 2010). August 1-6, 2010 Singapore, IFMBE Proceedings; Springer, 2010; Vol. 31, p 1000 10.1007/978-3-642-14515-5_255.

[49] Vyas R. N., Wang B. (2010). Electrochemical Analysis
of Conducting Polymer Thin Films. Int. J. Mol.
Sci..

[50] Ruano G., Iribarren J. I., Pérez-Madrigal M. M., Torras J., Alemán C. (2021). Electrical and Capacitive Response of Hydrogel Solid-like
Electrolytes for Supercapacitors. Polymers.

[51] Zhang W., Feng P., Chen J., Sun Z., Zhao B. (2019). Electrically
Conductive Hydrogels for Flexible Energy Storage Systems. Prog. Polym. Sci..

[52] Wang Y.-F., Sekine T., Takeda Y., Yokosawa K., Matsui H., Kumaki D., Shiba T., Nishikawa T., Tokito S. (2020). Fully Printed PEDOT:PSS-Based Temperature
Sensor with
High Humidity Stability for Wireless Healthcare Monitoring. Sci. Rep..

[53] Honda W., Harada S., Arie T., Akita S., Takei K. (2014). Wearable,
Human-Interactive, Health-Monitoring, Wireless Devices Fabricated
by Macroscale Printing Techniques. Adv. Funct.
Mater..

[54] Vuorinen T., Niittynen J., Kankkunen T., Kraft T. M., Mantysalo M. (2016). Inkjet-Printed
Graphene/PEDOT:PSS Temperature Sensors on a Skin-Conformable Polyurethane
Substrate. Sci. Rep..

[55] Bali C., Brandlmaier A., Ganster A., Raab O., Zapf J., Hübler A. (2016). Fully Inkjet-Printed Flexible Temperature Sensors Based
on Carbon and PEDOT:PSS. Mater. Today: Proc..

[56] Hong S. Y., Lee Y. H., Park H., Jin S. W., Jeong Y. R., Yun J., You I., Zi G., Ha J. S. (2016). Stretchable Active
Matrix Temperature Sensor Array of Polyaniline Nanofibers for Electronic
Skin. Adv. Mater..

[57] Garcia-Torres J., Colombi S., Mahamed I., Sylla D., Arnau M., Sans J., Ginebra M.-P., Alemán C. (2023). Nanocomposite
Hydrogel with Temperature Response for Capacitive Energy Storage. ACS Appl. Energy Mater..

[58] Puiggali-Jou A., Babeli I., Roa J. J., Zoppe J. O., Garcia-Amoros J., Ginebra M.-P., Aleman C., Garcia-Torres J. (2021). Remote Spatio-Temporal
Control of a Magnetic and Electroconductive Reversible Hydrogel Network
via Magnetic Fields: A New Concept in Soft Electronics. ACS Appl. Mater. Interfaces.

[59] Liu H., Li M., Ouyang C., Lu T. J., Li F., Xu F. (2018). Biofriendly,
Stretchable, and Reusable Hydrogel Electronics as Wearable Force Sensors. Small.

[60] Cunha I., Barras R., Grey P., Gaspar D., Fortunato E., Martins R., Pereira L. (2017). Reusable Cellulose-Based
Hydrogel
Sticker Film Applied as Gate Dielectric in Paper Electrolyte-Gated
Transistors. Adv. Funct. Mater..

