# Supplementary material for: Soft, Sustainable, and Sensitive: Biopolymer-Based Hydrogels as Recyclable Temperature Sensors for Skin-Integrated Electronics
Source: ACS Appl Bio Mater. 2025 Nov 13;8(12):10890–902. doi: 10.1021/acsabm.5c01607 (PMC12709615; doi:10.1021/acsabm.5c01607)
Supplement: Supplementary file 1 [file mt5c01607_si_001.pdf]

SUPPORTING INFORMATION

Soft, Sustainable, and Sensitive: Biopolymer-based Hydrogels as Recyclable Temperature Sensors for Skin-Integrated Electronics

David Naranjo,[a,b,c] Juan Torras,[a,c] Jose García-Torres,[b,c,d,*]

[a] *IMEM-BRT Group, Departament d'Enginyeria Química, EEBE, Universitat Politècnica de Catalunya, C/ Eduard Maristany, 10-14, Ed. I, 2nd floor, 08019, Barcelona, Spain*

[b] *Biomaterials, Biomechanics and Tissue Engineering Group, Department of Materials Science and Engineering, Escola d'Enginyeria Barcelona Est (EEBE) and Institute for Research and Innovation in Health (IRIS), Universitat Politècnica de Catalunya (UPC), 08019, Barcelona, Spain*

[c] *Barcelona Research Center in Multiscale Science and Engineering, Universitat Politècnica de Catalunya, 08019 Barcelona, Spain*

[d] *CIBER en Bioingeniería, Biomateriales y Nanomedicina, CIBER-BBN, Zaragoza 50018, Spain*

*jose.manuel.garcia-torres@upc.edu

ORCID's:

D. Naranjo: 0000-0001-9555-8397

J. Torras: 0000-0001-8737-7609

J. Garcia-Torres: 0000-0002-3996-0274

Table S1. Volume of PEDOT:PSS (1.3% w/w) dispersion used to synthesize each gel sample.

Hydrogel	Volume of PEDOT:PSS Dispersion (mL)
Chit/Ag	0.00
Chit/Ag/PEDOT:PSS(5)	0.95
Chit/Ag/PEDOT:PSS(10)	2.00
Chit/Ag/PEDOT:PSS(20)	4.50
Chit/Ag/PEDOT:PSS(30)	7.75

Table S2. Additional pore metrics computed from SEM images

Hydrogel	Number of pores processed	ECD (μm)	Area (μm^2)	Perimeter (μm)	Circularity	Feret max (μm)	Feret min (μm)	Aspect ratio
Chit/Ag	1899	41.3 ± 31.9	2902.7 ± 1477.9	239.6 ± 176.2	0.5 ± 0.2	72.1 ± 25.8	39.1 ± 30.1	2.0 ± 0.9
Chit/Ag/PEDOT:PSS(5)	1883	44.6 ± 38.3	3052.5 ± 2270.4	245.2 ± 231.9	0.6 ± 0.2	75.6 ± 27.8	41.5 ± 36.0	2.0 ± 0.9
Chit/Ag/PEDOT:PSS(10)	1793	46.0 ± 33.3	3954.7 ± 3128.4	263.2 ± 130.3	0.5 ± 0.2	81.8 ± 42.9	41.8 ± 36.4	2.1 ± 1.0
Chit/Ag/PEDOT:PSS(20)	1866	49.2 ± 38.1	3643.7 ± 3400.9	309.2 ± 229.9	0.5 ± 0.2	91.5 ± 42.2	43.2 ± 40.6	2.2 ± 1.0
Chit/Ag/PEDOT:PSS(30)	1443	52.7 ± 43.4	4769.4 ± 2663.7	315.4 ± 271.3	0.5 ± 0.2	91.1 ± 55.2	49.7 ± 40.4	2.0 ± 1.1

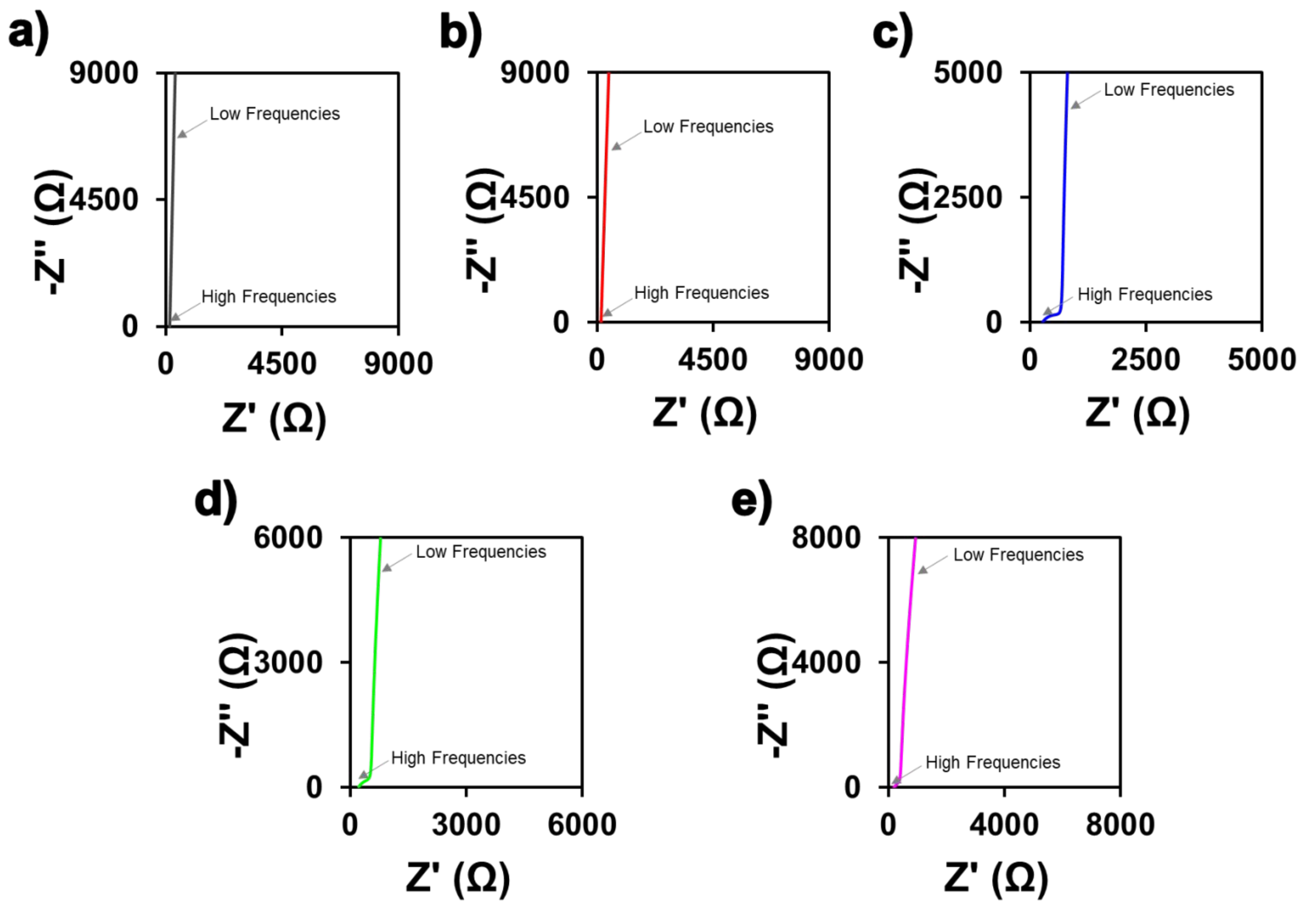

Figure S1. Nyquist plot of a) Chit/Ag, b) Chit/Ag/PEDOT:PSS(5), c) Chit/Ag/PEDOT:PSS(10), d) Chit/Ag/PEDOT:PSS(20), and e) Chit/Ag/PEDOT:PSS(30) hydrogels showing the low and high frequency limits.

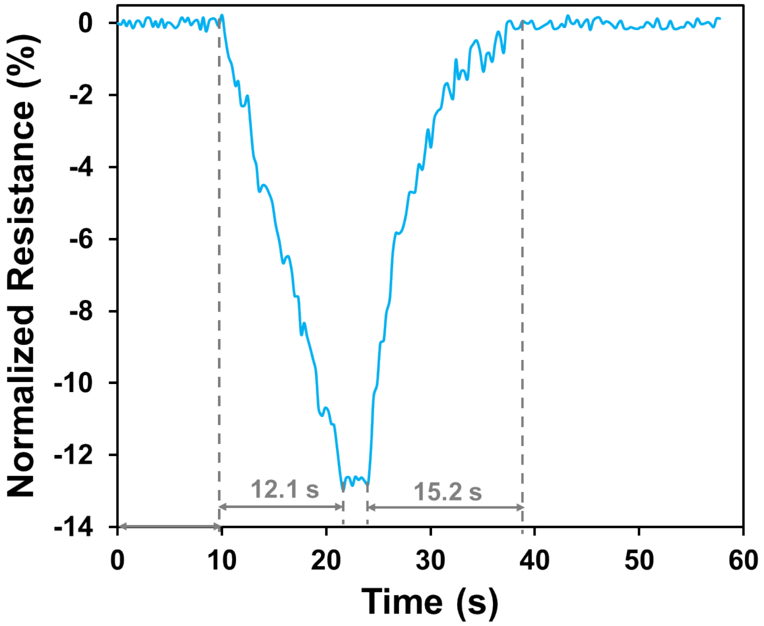

Figure S2. Time window showing the response and recovery of Chit/Ag/PEDOT:PSS(20) film from room temperature $T_0 \sim 23$ ºC to 37 ºC.

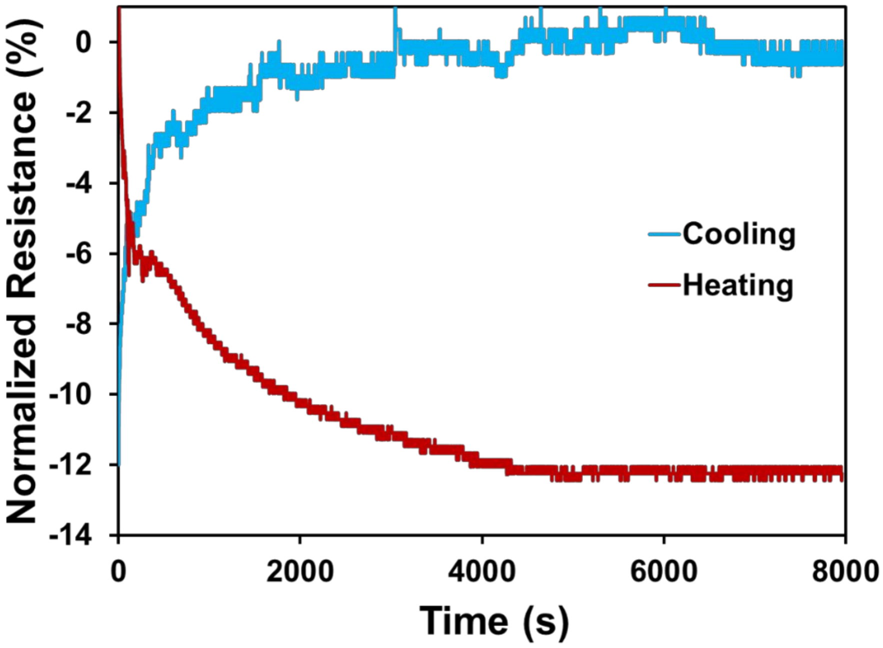

Figure S3. Cell culture media continuous temperature monitoring.

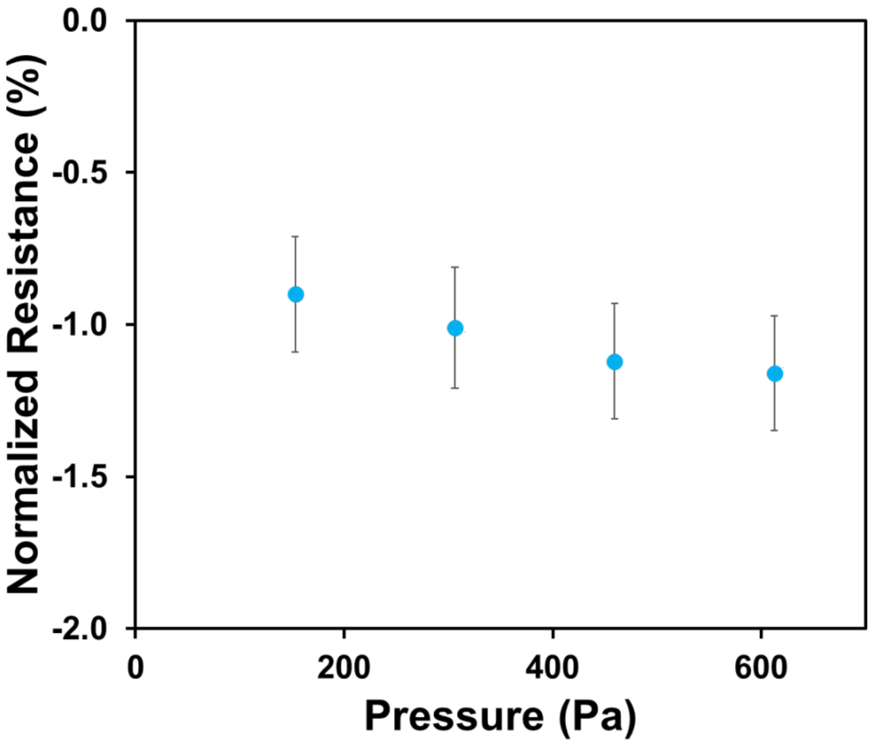

Figure S4. Response to pressure of Chit/Ag/PEDOT:PSS(20) film at room temperature $T_0 \sim 23$ ºC.
